# Editorial: Current perspectives on *Pseudomonas aeruginosa*: epidemiology, virulence and contemporary strategies to combat multidrug-resistant (MDR) pathogens

**DOI:** 10.3389/fmicb.2022.975616

**Published:** 2022-07-26

**Authors:** Payam Behzadi, Cecilia Ambrosi, Daniela Scribano, Stefania Zanetti, Meysam Sarshar, Márió Gajdács, Matthew Gavino Donadu

**Affiliations:** ^1^Department of Microbiology, College of Basic Sciences, Shahr-e-Qods Branch, Islamic Azad University, Tehran, Iran; ^2^Department of Human Sciences and Promotion of the Quality of Life, San Raffaele Open University, Scientific Institute for Research, Hospitalization and Healthcare (IRCCS), Rome, Italy; ^3^Department of Public Health and Infectious Diseases, Sapienza University of Rome, Rome, Italy; ^4^Department of Biomedical Sciences, University of Sassari, Sassari, Italy; ^5^Research Laboratories, Bambino Gesù Children's Hospital, Scientific Institute for Research, Hospitalization and Healthcare (IRCCS), Rome, Italy; ^6^Department of Oral Biology and Experimental Dental Research, Faculty of Dentistry, University of Szeged, Szeged, Hungary; ^7^Hospital Pharmacy, Azienda Ospedaliero Universitaria di Sassari, Sassari, Italy

**Keywords:** *Pseudomonas aeruginosa*, multidrug resistance, quorum sensing, *in vitro*, infectious disease

*Pseudomonas aeruginosa* is a member of *Pseudomonatoda* (synonym *Proteobacteria*) phylum, belonging to the group of non-fermenting Gram-negative bacteria (NFGNB) (Oren and Garrity, 2021). Members of the NFGNB are similar in that they are not able to use fermentative biochemical processes to provide to their biological fuel from sugars for vital activities (McGowan, 2006). Currently, the *Pseudomonas* genus contains 299 different species (https://lpsn.dsmz.de/genus/pseudomonas). Among them, *P. aeruginosa* has a considerable capability for adapting to many different environments through the accessibility to a wide range of metabolic pathways. Furthermore, the presence of numerous virulence factors in its arsenal—owing to its high genetic plasticity—enforces the pathogenicity of *P. aeruginosa* (Rumbaugh, 2014). The recognition of a versatile range of resistance mechanisms in these motile and non-fastidious bacteria has made it a great concern for healthcare institutions worldwide, in particular among immunocompromised and hospitalized patients, associated with invasive therapeutic procedures; the treatment of *P. aeruginosa* infections are becoming increasingly challenging to clinicians, due to the increasingly limited therapeutic choices. Due to the many acquired and intrinsic resistance mechanisms (which are both relevant in the overall resistant profile of *P. aeruginosa*) multi-drug resistant (MDR) strains of this species are now part of the everyday clinical practice (Gajdács et al., 2021b). Various factors and characteristics comprising genomic plasticity, cell-mediated factors, pigments, toxins and protease enzymes, extensive biofilm-formation, secretion systems, adaptive mechanisms, quorum sensing (QS), hypermutable strains, overexpressing efflux pumps, OprD porin deficient mutants, inactivating enzymes acting on both antimicrobials and immune system, target alternations, and the presence of persisters make *P. aeruginosa* one of the most effective pathogens in the field of medical microbiology (Behzadi et al., 2021).

Due to the importance of the topic, we designed a Research Topic in Frontiers in Microbiology to collect interesting and outstanding papers, to address the virulence, drug resistance and management strategies associated with *P. aeruginosa* and other members of the genus. To our great delight, eight thematic articles, corresponding to 55 authors, were published by established scientists around the world in our Research Topic, highlighting the recent data and information regarding current perspectives on *P. aeruginosa*. Cervoni et al. have studied the effects of exogenous and endogenous phosphoethanolamine (PEtN) transferase enzymes on colistin resistance and fitness in *P. aeruginosa*. Indeed, mutagenic modifications are common both in the chromosomal (endogenous) gene of *eptA* in *P. aeruginosa* and in the plasmid borne (exogenous or mobilized) resistance-determinant gene of *mcr-1* in members belonging to the Enterobacterales order. Cervoni et al. showed that both proteins of EptA and MCR-1 likewise represent PEtN transferase activity by expression of associated genes cloned within a mobile genetic element of a plasmid in *P. aeruginosa*. Moreover, the expression of EptA and MCR-1 resulted in the same colistin resistance characteristics as it occurs through a lipid A-aminoarabinosylation process. As the conclusion of their interesting work, Cervoni et al. demonstrated that, although PEtN and MCR-1 increase the phenotypic resistance against colistin in *P. aeruginosa*, they affect neither on bacterial cell growth homeostasis nor cell envelope homeostasis in this pathogen.

In another invaluable paper published by Martínez-Alcantar et al. the bacterial adaptability and pathogenicity of the *P. aeruginosa* strain PAO1 was considered as their target goal. In this regard, they investigated the *fadD* paralogous genes, which are involved in bacterial pathogenicity and fatty acids' digestion in *P. aeruginosa* PAO1. In particular, they focused their attention to the gene of *fadD4*, analyzing the possible role in fatty acid and acyclic terpene degradation and bacterial pathogenicity. As an interesting result, Martínez-Alcantar et al. could find out that a terpenoyl-CoA-synthetase is expressed by *fadD4*, which contributes to fatty acid and acyclic terpene assimilation. Furthermore, Martínez-Alcantar and her team analyzed the fatty acid methyl ester belonging to bacterial lipopolysaccharide (LPS) in *P. aeruginosa* PAO1. Their results showed that mutated *fadD4* results in various changes in some parts of fatty acid and LPS molecules on lipid A as well. The *in vivo* investigations on the worm model of *Caenorhabditis elegans* and in rat models revealed the effective correlations between *fadD* genes and the bacterial pathogenicity via modifications of lipid A. In other words, FadD4 effectively contributed to LPS biosynthesis through the structural modification of lipid A. As an important virulence factor in Gram-negative bacteria, LPS of *P. aeruginosa* PAO1 may lead to expression of immune responses (including interleukins [ILs] 8 and 1ß and the glycoprotein of Toll-like receptor [TLR] 4 in the rat model).

Thees et al. have studied a small molecular weight bacterial metallothionein (MT) in *P. aeruginosa* PAO1, known as PmtA, a cysteine-rich protein belonging to the MT family. The role of PmtA was previously recognized as an effective virulence factor in biofilm formation and in facilitation of chronic infections *in vivo*. Although some similarities between metallothionein proteins from bacteria and eukaryotes—including antioxidant chemistry, sequence homology and the capability of heavy metal-binding site and eukaryotes—Thees et al. aimed at studying the importance of bacterial metallothionein proteins in bacterial virulence and infection. To achieve this, Thees et al. employed a PmtA-deficient (Δ*pmtA*) mutant of *P. aeruginosa* strain PAO1. They found that the expression of pyocyanin is associated with expression of PmtA. Moreover, the protective role of PmtA against oxidative stress may also support the production of pyocyanin. Interestingly, Thees et al. have also shown that PmtA is involved in bacterial biofilm formation, antibiotic resistance (against e.g., ciprofloxacin and cefepime) and bacterial virulence in a waxworm model. In summary, Thees et al. have shown in their interesting investigation that PmtA directly affects the expression of bacterial virulence factors in *P. aeruginosa* PAO1, thereby suggesting that PmtA should be recognized as a potential therapeutic target in the future for therapeutic prospects of *P. aeruginosa* PAO1 infections.

Another interesting paper published by Zhang et al. investigates the synergistic effects of a colistin-mefloquine combination against colistin resistant strains of *P. aeruginosa in vitro* and *in vivo* (in a *Galleria mellonella* infection model). As we know, the inappropriate use of antibiotics globally has led to a significant increase in MDR strains, including *P. aeruginosa*. The results in this study clearly showed that the combination of colistin-mefloquine had a synergistic effect against colistin resistant strains through inhibition of biofilm formation and matured biofilm elimination. Hence, the combination of colistin and mefloquine may be recognized as an effective potential in therapeutic procedures against infections caused by colistin resistant strains of *P. aeruginosa*. The study by Di Bonaventura et al. aimed at comparing the anti-biofilm, anti-virulence and anti-bacterial effects of two aminoglycosides, namely tobramycin and apramycin (a veterinary antibiotic) against *P. aeruginosa* strains involved in cystic fibrosis infections *in vitro*. In this study, the authors have shown that the MIC_90_ and MBC_90_ of apramycin were 4-fold lower than for tobramycin. Both of the antibacterials tested have bactericidal effects and were effective against mature bacterial biofilms with no cytotoxic effects. Although tobramycin and apramycin indicated no cytotoxic effects in IB3-1 bronchial epithelial cystic fibrosis cells, the application of these antibiotics has inductive effects on the overexpression of the MDR efflux pump *mexA* and *mexC* genes. Moreover, the use of tobramycin may also lead to increased expression of two genes, i.e., *aprA* and *toxA* encoding for the alkaline protease and the exotoxin A, respectively, possibly upregulating the virulence of *P. aeruginosa*. However, the rapid bacterial killing and the lack of observed resistance makes apramycin a candidate to be further evaluated in the treatment of *P. aeruginosa* infected CF patients

As aforementioned, QS is known as a critical virulence factor in *P. aeruginosa*, and an important regulatory mechanism based on the population-density of bacteria. In this regard, Collalto et al. studied on the effects of clofoctol and niclosamide as anti-virulence drug on QS-signal production and the *P. aeruginosa in vitro* susceptibility. The results of this investigation indicated that the effects of clofoctol and niclosamide on bacterial QS inhibition were highly variable. Interestingly, Collalto et al. have shown that clofoctol had a broader inhibitory effect on bacterial QS on isolates from patients with cystic fibrosis in comparison with niclosamide. Moreover, colfoctol may lead to the decreased expression of pyocyanin (a virulence factor and pigment of *P. aeruginosa*) as an outcome resulted from QS inhibition.

In their interesting study, Kiyaga et al. through the whole-genome sequencing approach, aimed to perform a phylogenetic analysis, sequencing typing, and genome characterization of *P. aeruginosa* isolates collected from different clinical specimens. They also found a new MDR clone carrying a plethora of virulence genes. In their paper, Kiyaga et al. have described 12 new lineages of *P. aeruginosa*. Overall, the sequence type 3674 (ST3674), as a new MDR clone, was carrying the highest number of virulence genes and extensive levels of antimicrobial resistance. Finally, in a Perspective article by Hart and Morici, the authors described the significance of a potential vaccine against *P. aeruginosa*, and they have reviewed the literature of the last two decades over the landscape of potential (pre-clinical and clinical) prophylactic vaccine candidates. Their paper highlights that such a vaccine would decrease our dependence on antibiotics, and would significantly reduce the burden of these infections, especially in healthcare-associated infections and the more severe bacteremia/sepsis (Hart and Morici).

Antimicrobial resistance and the “renaissance” of infectious diseases being important factors of morbidity and mortality are critical public health issues, which will define the fate of healthcare in the next decades. *P. aeruginosa* is one of the critical priority pathogens causing serious concern, due to its adaptability, versatility in survival and high rates of resistance (Gajdács et al., 2021a). The current Research Topic highlights articles originating from different study groups that summarize the recent advancements in the fight against *P. aeruginosa;* we do hope that the articles published in the topical collection will be considered as important findings for other scientist involved in similar research to aid the development of novel antimicrobial strategies.

## Author contributions

PB, CA, DS, SZ, MS, MG, and MD have contributed to the writing of the initial version of the manuscript. All authors have read and agreed to the final version of the manuscript.

## Funding

MG was supported by the János Bolyai Research Scholarship (BO/00144/20/5) of the Hungarian Academy of Sciences. The research was supported by the ÚNKP-21-5-540-SZTE New National Excellence Program of the Ministry for Innovation and Technology from the source of the National Research, Development and Innovation Fund. MG would also like to acknowledge the support of ESCMID's 30 under 30 Award. The research was also supported by Bandi Ateneo Sapienza, grant number RP120172B7FF9E6F to CA Salary of DS was supported by POR Lazio FSE 2014-202O and Sapienza Ateneo fundings. Salary of MS was supported by the Italian Ministry of Health (starting grant SG-2018-12365432).

## Conflict of interest

The authors declare that the research was conducted in the absence of any commercial or financial relationships that could be construed as a potential conflict of interest.

## Publisher's note

All claims expressed in this article are solely those of the authors and do not necessarily represent those of their affiliated organizations, or those of the publisher, the editors and the reviewers. Any product that may be evaluated in this article, or claim that may be made by its manufacturer, is not guaranteed or endorsed by the publisher.
